# Machine learning predicts improvement of functional outcomes in traumatic brain injury patients after inpatient rehabilitation

**DOI:** 10.3389/fresc.2022.1005168

**Published:** 2022-09-22

**Authors:** Irene Say, Yiling Elaine Chen, Matthew Z. Sun, Jingyi Jessica Li, Daniel C. Lu

**Affiliations:** ^1^ Department of Neurosurgery, David Geffen School of Medicine, University of California, Los Angeles, CA, United States; ^2^ Department of Statistics, University of California, Los Angeles, CA, United States; ^3^ Neuromotor Recovery and Rehabilitation Center, David Geffen School of Medicine, University of California, Los Angeles, CA, United States; ^4^ Brain Research Institute, University of California, Los Angeles, CA, United States

**Keywords:** machine learning, artificial intelligence, functional independence measure, prediction model, traumatic brain injury, rehabilitation

## Abstract

Survivors of traumatic brain injury (TBI) have an unpredictable clinical course. This unpredictability makes clinical resource allocation for clinicians and anticipatory guidance for patients difficult. Historically, experienced clinicians and traditional statistical models have insufficiently considered all available clinical information to predict functional outcomes for a TBI patient. Here, we harness artificial intelligence and apply machine learning and statistical models to predict the Functional Independence Measure (FIM) scores after rehabilitation for traumatic brain injury (TBI) patients. Tree-based algorithmic analysis of 629 TBI patients admitted to a large acute rehabilitation facility showed statistically significant improvement in motor and cognitive FIM scores at discharge.

## Introduction

Traumatic brain injury (TBI) is a leading cause of death and disability in America (1). TBI represents a continuum of different types of brain injury, ranging from a mild concussion to life-threatening blunt and penetrating head injury (2). Mild to moderate TBI may lead to headache, a transient loss of consciousness, memory loss, and confusion, while severe TBI creates severe disability, such as confusion, weakness, comatose states, and even death. Physiologically, the brain is subject to local mass effect, stretch/shear/rotational forces with diffuse axonal injury, increased intracranial pressure from hemorrhage, contusion, and skull fracture, and is exquisitely sensitive to generalized, secondary insults including hypotension and hypoxia. Marginalized populations, including ethnic minorities, the homeless, and those in rural communities, are disproportionately affected. Suicide, falls, motor vehicle accidents, and assaults consistently account for greater than 200,000 TBI-related hospitalizations yearly (3). While severe TBI can be fatal, survivors living with mild to moderate TBI experience a broad spectrum of unpredictable short and long-term physical and mental disabilities that compromise their quality of life and productivity. These patients require extensive social and financial support to manage several chronic diseases. This ranges from immediately life-threatening conditions such as epilepsy, substance abuse, diabetes, cardiopulmonary disease, depression, and stroke to conditions that reduce quality of life such as chronic pain, mood disorders, insomnia, and cognitive slowing (4, 5). For example, patients with moderate-severe TBI have reduced life expectancy, shortened by 7–9 years compared to age-matched controls (6). Furthermore, five years after injury, 1 in 5 TBI patients is either dead, disabled, unemployed, or lives in a nursing home, and this statistic has not changed in the last decade (5). Nationwide, the latest Center for Disease Control (CDC) report estimates the direct aggregate medical costs of TBI at over $70 billion, without accounting for years of lost or reduced productivity and unemployment (2). Cost notwithstanding, we are unable to reliably identify those who will benefit most from valuable rehabilitation services or tailor an individualized treatment plan to maximize recovery. Current clinical practice is subjective, anecdotal, and falls short of incorporating all available clinical data.

Each patient and their specific brain injury are uniquely multifaceted, posing challenges in predicting the long-term outcomes of any single treatment. Countless permutations of anatomic and physiologic mechanisms of traumatic brain injury account for the heterogeneity in clinical phenotype. These mechanisms can vary from direct blunt contact to penetrating brain injury. Widely accepted clinical outcome measures such as the Glasgow Outcome Score (GOS, GOS-E), Disability Rating Scale (DRS), and the Coma Recovery Scale (CRS) standardize communication by calculating patient clinical data and documenting progress but fail to provide the prognostic granularity sought after by patients and their families (6–8). Clinical prediction models derived from the Corticosteroid Randomization after Significant Head Injury (CRASH) and the International Mission on Prognosis and Analysis of Clinical Trials in TBI (IMPACT) trials incorporate patient clinical data, imaging, and laboratory values, all in pursuit of predicting patient outcomes (9). However, studies seeking external validation of these models found only 75%–87% accuracy in predicting mortality or unfavorable outcomes and have limited generalizability. In other words, this leaves an unacceptably high degree of uncertainty in predicting outcomes for at least 1 in 4 patients (10–15). This statistical inaccuracy may be explained by the limited quantity and quality of descriptive clinical data points incorporated.

Prediction algorithms developed by leading statisticians have transformed modern-day medical research using machine learning. These sophisticated algorithms “learn” from large patient datasets to predict outcomes and guide decision-making (16). Machine learning, which surpasses the average human capacity for data interpretation, has the unique potential to drive precision medicine and forecast outcomes based on known permutations of individual patient data points (17). In fact, combining machine learning with traditional logistic regression models has generated reliable, highly accurate predictions in several neurosurgical arenas, ranging from recurrent lumbar disc herniations, surgical site infections, readmissions, and complications after tumor resection (18–23). However, little research exists on the application of machine learning to predicting outcomes in traumatic brain injury.

In this study, we collaborated with data scientists and expert statisticians to apply advanced machine learning and statistical modeling to predict functional independence measure (FIM) scores in a large cohort of traumatic brain injury patients completing a comprehensive rehabilitation program (24–26). We evaluate both the accuracy and fitness of traditional and machine learning statistical models to predict patient FIM scores. Our objective is to systematically assess the impact of inpatient rehabilitation on functional independence for traumatic brain injury patients. We hypothesize that quantifying the influence of rehabilitation on specific functional independence measures may guide future resource allocation and optimize decision-making during rehabilitation.

## Materials and methods

### Study design

Retrospective analysis of a prospectively collected data set of all traumatic brain injury patients admitted to a single acute rehabilitation facility (Casa Colina Acute Rehabilitation Unit, Pomona, CA) between 2010 and 2015 identified 629 patients. Pediatric, pregnant, and deceased patients were excluded from the study. Target inclusion criteria included adult patients with traumatic brain injury requiring inpatient rehabilitation after hospital discharge. Documented functional independence measure (FIM) scores were obtained at the time of admission and upon discharge. The Institutional Review Board (IRB) at the University of California, Los Angeles (UCLA) has exempted our study from its formal review, given the study's retrospective nature, under IRB #15-001380.

### Statistical analysis

The Functional Independence Measure (FIM) is a widely accepted, 18-item motor and cognitive function score that originated from the American Academy of Physical Medicine and Rehabilitation and the American Congress of Rehabilitation Medicine (27, 28). The FIM is calculated as a composite score based on direct observation by a multidisciplinary rehabilitation team that documents the patient's disability and level of assistance required to perform activities of daily living (29). Each motor and cognitive item earns an ordinal numerical score ranging from 1 to 7. The motor-FIM categories include eating, grooming, bathing, dressing the upper or lower body, toileting, bladder control, bowel control, bed transfer, toilet transfer, tub transfer, walk/wheelchair, and stairs. The cognitive-FIM categories include comprehension, expression, social interaction, problem-solving, and memory. Typically, a score of 1 means total assistance and 7 means full independence.

We first examined the efficacy of rehabilitation by testing whether the FIM scores at discharge improved compared to the FIM scores at admission. Specifically, we conducted paired t-tests for each of the 18 items. Then, we adjusted for multiple testing using the Bonferroni procedure. We applied established statistical and novel machine learning algorithms to predict patients' FIM scores at the end of rehabilitation. Our responses of interest are patients' FIM scores at discharge, each of which is an ordinal variable that takes integer values from 1 to 7. An ordinal variable differs from either a categorical variable because of its ordering (e.g., 1 < 2 < 23 … <7) and differs from a continuous variable because of its discrete nature (i.e., it cannot take on values such as 1.5). Although both the statistics and the machine learning communities have developed various prediction methods, most of them are tailored to continuous or categorical responses. Ordinal regression is a statistical method designed to handle ordinal responses by considering their ordering and discreteness. Tree-based machine learning algorithms designed to predict continuous responses are capable of handling ordinal responses if we round the predicted value to the nearest integer (30).

Input features included 40 predictors including demographic information, diagnostic characteristics, comorbidities, FIM scores at admission, length of stay, etc. as seen in Table 1. Continuous predictors include age and length of stay at the rehabilitation center. Categorical predictors include gender (male; female), diagnosis (cerebral contusion; concussion; intracranial hemorrhage (ICH): subarachnoid hemorrhage (SAH), subdural hematoma (SDH), epidural hematoma (EDH)), brain tumor (yes; no), diabetes (yes; no), other cognitive impairment/dementia (yes; no), coronary artery disease (yes; no). These categorical predictors were selected as the leading significant types of traumatic brain injury and to remove potential confounding factors, such as the presence of a brain tumor or pre-existing cognitive impairment. Ordinal predictors include FIM scores and functional modified FIM scores at admission. Data on predictors and responses were collected by independent, trained data collectors.

**Table 1:** Mean and standard deviation of input features used in all predictive modelling.

Predictors	Mean (sd)	Predictors	Mean (sd)
Gender	NA	AdmitFIMBladderCtrl	2.75 (2.25)
PreHospitalLivingSetting	NA	AdmitFIMBowelCtrl	4.2 (2.26)
PreHospitalLivingWith	NA	AdmitFIMBedTransfer	2.75 (1.23)
Diagnosis	NA	AdmitFIMToiletTransfer	2.75 (1.27)
brain.tumor	NA	AdmitFIMTubTransfer	2.09 (1.91)
DM	NA	AdmitFIMWalkWheelchair	1.96 (1.35)
Other.dementia	NA	AdmitFIMStairs	1.02 (1.32)
Coronary.artery.disease	NA	AdmitFIMComprehension	3.86 (1.47)
AdmitFIMWalkWheelchairMeasured	NA	AdmitFIMExpression	3.82 (1.62)
AdmitFIMComprehensionMeasured	NA	AdmitFIMSocialInteraction	4.04 (1.71)
AdmitFIMExpressionMeasured	NA	AdmitFIMProblemSolving	2.99 (1.37)
LOS	18.7 (12.8)	AdmitFIMMemory	3 (1.34)
AGE	62.13 (23.47)	AdmitFnModBladderLvlAssist	2.76 (2.26)
AdmitSwallowingStat	2.42 (0.71)	AdmitFnModBladderFreqAccidents	5.73 (1.02)
AdmitFIMEating	4.16 (1.73)	AdmitFnModBowelLvlAssist	4.21 (2.27)
AdmitFIMGrooming	3.8 (1.56)	AdmitFnModBowelFreqAccidents	5.95 (0.59)
AdmitFIMBathing	2.65 (1.54)	AdmitFnModDistWalked	1.68 (0.95)
AdmitFIMDressingUpper	3.46 (1.45)	AdmitFnModDistWheelchair	1.61 (0.99)
AdmitFIMDressingLower	2.57 (1.29)	AdmitFnModWalk	1.94 (1.36)
AdmitFIMToileting	2.07 (1.32)	AdmitFnModWheelchair	1.92 (1.5)

Input features include basic demographic information, pre-existing conditions, and pre-admission Functional Independence Measure (FIM) scores.

**Figure 1:**
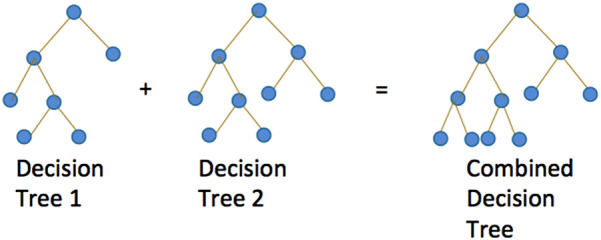
Graphical illustration of tree-based algorithms such as random forests and XGBoost. Both algorithms build multiple trees for prediction. Random forests build independent trees whereas XGBoost builds trees based on the performance of previous trees.

By using the above features as predictors and each FIM item at discharge as the response, we applied the following eight algorithms to our prediction analyses: statistical methods, including parallel or semi-parallel ordinal regression with lasso, ridge, or elastic net penalty and machine learning methods including tree-based algorithms such as XGBoost and random forests (31–34). Figure 1 graphically illustrates the concept of tree-based algorithms, featuring the building of innumerable tree branches and decision nodes of the sample population to ultimately predict those same features. Our objective was to find the best prediction algorithm that minimizes error. We chose not to include neural networks; because the assignment of the FIM scores involved subjective judgment from medical providers, neural networks could easily overfit the data given the high level of noise in the data. The semi-parallel ordinal regression models are more flexible than parallel models and prone to overfitting.

We evaluated the prediction accuracy of statistical and machine learning methods using five-fold cross-validation. Hyperparameters were tuned again by five-fold cross-validation using only the training set. The algorithms were subsequently trained with optimized hyperparameters on the full training set and evaluated on the test set. We evaluated predictive accuracy by computing the L1 loss, defined as the absolute difference between the true outcome and the predicted outcome averaged over patients. For benchmarking, we used the FIM scores at admission as the predicted value at discharge, which we referred to as the baseline approach. We implemented ordinal regression in R using R package ordinalNet (version 2.9), random forests using R package randomForest (version 4.6-14), and XGBoost using R package xgboost (32–35). (version 1.4.1.1).

**Figure 2:**
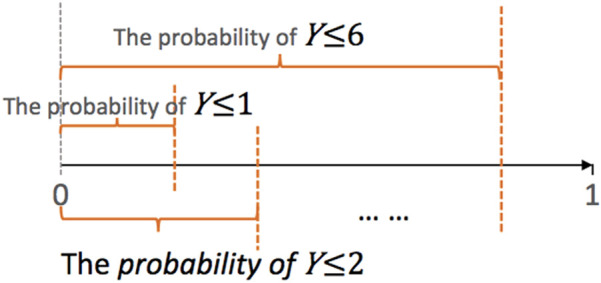
Graphical illustration of ordinal regression. Suppose the class labels range from 1 to 7. The ordinal regression trains 6 logistic models to predict the probability of *Y* ≤ *j*, *j* = 1,…,6*.* The parallel ordinal regression model assumes that these 6 sub-models share the same set of coefficients, whereas the semi-parallel model forces the (K-1) sets of coefficients to be similar and close to 0.

When applied to predicting FIM scores that range from 1 to 7, the ordinal regression trains 6 logistic regression sub-models to compute the probabilities of a score no greater than 1, 2, …, 6, respectively (Figure 2). Given a new datapoint with unknown score Y, the trained ordinal regression computes the probability of Y=i, for example, as the probability of Y≤i minus the probability of Y≤i−1. Then the model outputs the score with the highest probability as the predicted value of this datapoint.

To increase the stability of the final model, we added LASSO, elastic net, or ridge penalty to the training of individual sub-models. In addition, we also forced the trained parameters of 6 sub-models to share various levels of similarity. The parallel ordinal regression forces the 6 sub-models to share the same set of coefficients except for the intercept. The nonparallel ordinal regression imposes no such constraints and allows the coefficients to differ completely. The semi-parallel ordinal regression lies between the parallel and the non-parallel regression; it enforces the coefficients to be similar but not exactly the same. We applied all the three types of models, and only the parallel and semi-parallel models were successfully trained on our data. Therefore, the results of the non-parallel ordinal regression models are not shown.

## Results

**Figure 3:**
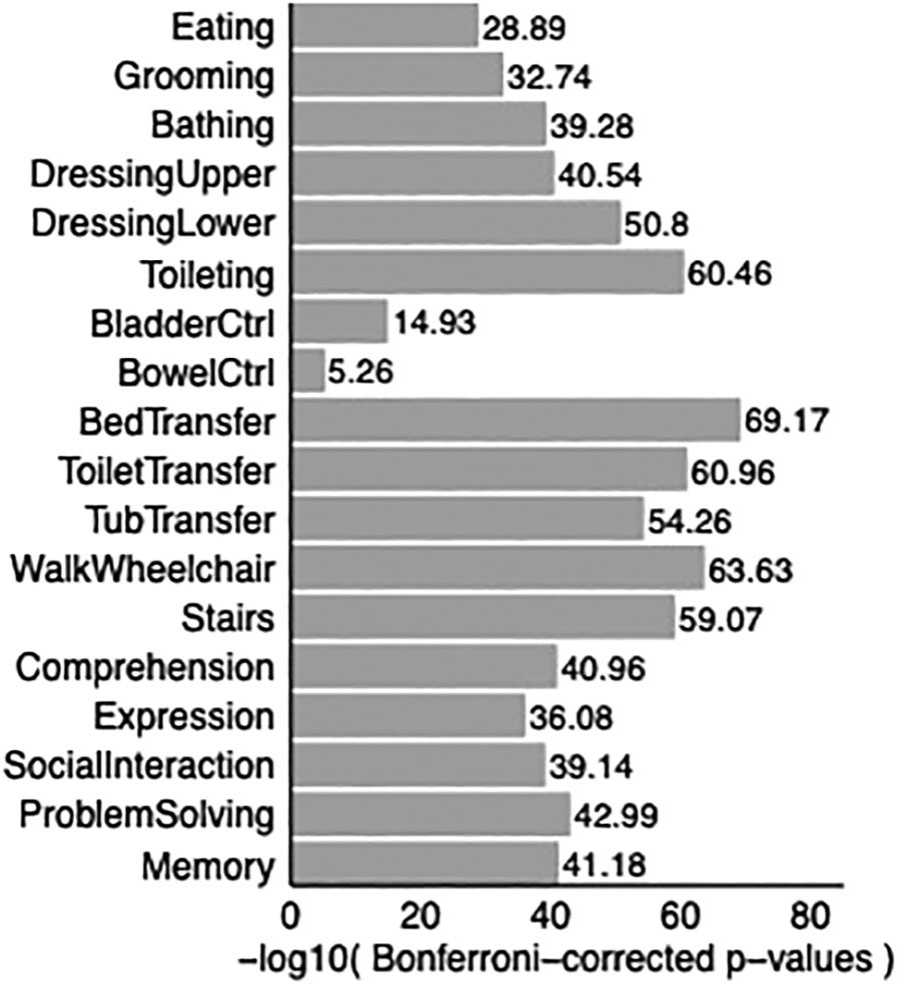
Bar graph shows *p*-values comparing FIM scores at discharge with FIM scores at admission. *P*-values are adjusted for multiple correction. All *p*-values are smaller than 0.05, showing improvement in all 18 Functional Independence Measurement (FIM) items upon discharge in TBI patients compared to admission to acute rehabilitation.

Paired t-tests demonstrated that rehabilitation showed statistically significant improvement in the functional independence of patients in all 18 items of FIM (Figure 3).

**Figure 4:**
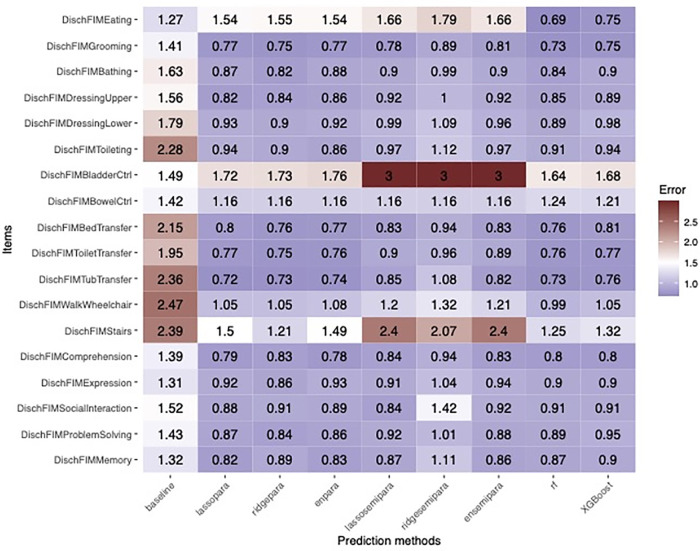
Heatmap showing the error rates of machine learning prediction algorithms across the 18 Functional Independence Measurement (FIM) scores in TBI patients. Statistical algorithms included parallel and semi-parallel ordinal regression with lasso, ridge, or elastic net penalty, random forest, and XGBoost.

**Figure 5:**
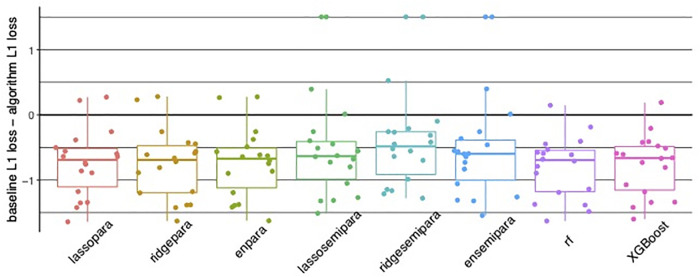
(**A**) Bar and whisker plot showing superior accuracy of tree-based algorithms, in random forest (rf) and XGBoost with controlled algorithm L1 loss compared to parallel and semi-parallel ordinal regression. (**B**) Bar graphs for individual Functional Independence Measure (FIM) items illustrating tree-based algorithms random forest (rf) and XGBoost with the lowest error.

Regarding prediction analysis, tree-based algorithms, including random forests and XGBoost, demonstrated the best overall accuracy. Specifically, tree-based algorithms offered the strongest advantage in 3 items: Eating, bladder control, and stairs, while achieving comparable accuracy in the remaining 15 items as visualized in Figure 4. Figures 5a,b recapitulate the superiority of tree-based algorithms through a box-whisker plot and bar graphs. For eating, only tree-based algorithms outperformed the baseline model by controlling L1 loss under 0.75, while the L1 loss of ordinal regressions, parallel or semi-parallel, all exceeded 1.5. In terms of Bladder Control, tree-based algorithms achieved the L1 loss of around 1.6, showing a slight advantage over parallel regressions (L1 loss: around 1.7) and an enormous lead over semi-parallel ordinal regressions (L1 loss: 3). Similarly, in terms of Stairs, tree-based algorithms achieved an L1 loss of around 1.25, similar to that of parallel ordinal regression with the ridge penalty; in contrast, semi-parallel ordinal regressions' L1 loss exceeded 2. Machine learning algorithms demonstrated an overall advantage over statistical methods.

## Discussion

Predicting the clinical outcome for those living with traumatic brain injury (TBI) is critical, carrying far-reaching implications for their families, health systems, and society. Yet, expert opinion and traditional statistical analyses have been unable to reliably incorporate the numerous clinical data points to accurately forecast a patient's outcome, reintegration into society, and ultimately, the precise financial resources required. Because the Functional Independence Measure (FIM) has emerged as an externally validated, comprehensive clinical score of a patient's level of disability, we focus on these points as surrogate markers of functional improvement (24, 36–39). We developed the first known investigation on predictive statistical modeling of Functional Independence Measure (FIM) scores for TBI patients in an acute rehabilitation facility. Our tree-based machine learning algorithms using random forest and XGBoost demonstrated a high degree of accuracy and predicted on average within ±1 from the true FIM scores for at least 14 items out of 18, showing promise for real-world applications.

Tree-based decision algorithms such as random forest and XGBoost demonstrated the highest degree of error for bowel/bladder control and stairs categories. Deterministic algorithms are notoriously sensitive to statistical noise and reflect the inherent variability within the data, such as during collection, entry, and interpretation. For example, at separate timepoints, FIM scores are determined by direct observation by one or multiple members of a multidisciplinary team of physicians, nurses, and physical/occupational therapists. Since FIM scores are designed to describe the burden of care required, a single FIM score of 7 for independent bladder control, for instance, may describe a patient who either voids normally, self-catheterizes, or is completely dependent on dialysis. Furthermore, grading may be nuanced and subject to the amount of time “reasonably expected” to complete any given task.

Semi-parallel ordinal regression algorithms showed lower accuracy than the other algorithms, most notably in eating, bladder control, and stairs. Because of their increased model flexibility compared to parallel ordinal regression, semi-parallel regression models severely overfit the data. This may be explained by the subjectivity of FIM scoring, resulting in unfavorable prediction accuracy.

High-quality statistical modeling and applications of machine learning to the traumatic brain injury population after hospital discharge are sparse and concentrate mostly on predicting in-hospital mortality. Satyadev et al. similarly recognized the insufficiency of traditional prognostic calculators and ultimately selected a random forest model to predict hospital discharge disposition after traumatic brain injury (40). Tu et al. applied several statistical models, but found that a logistical regressional model best predicted mortality of traumatic brain injury patients triaged in an emergency room (41). Similar to our findings, Warman et al. found that another tree-based model, XGBoost predicted in-hospital mortality for high-income and low-income countries, compared to clinical prognostic calculators CRASH and IMPACT (42). To date, there is limited evidence analyzing statistical modeling for traumatic brain injury patients after hospital discharge, leaving considerable uncertainty in predicting their functional outcomes.

Our study is limited by our moderate sample size of 629 patients and reflects patients at a single inpatient rehabilitation center in southern California. Specifically, it is not completely generalizable to all TBI patients and captures only the subset who have the resources to enter and stay in a rehabilitation facility. Selection to enter inpatient rehabilitation is based on a number of factors, namely a patient's severity of injury, insurance status, and level of family support.

Our study's greatest limitation likely lies within the gradient of the clinical pathology itself. The unique combination of patient factors, mechanism of injury, and resulting clinical phenotype can only be approximated by advanced statistics, but never completely defined. With further refinement of the models and the data, it is plausible that machine learning algorithms could be used in practice.

Future study could expand to multiple centers, capturing a diversity of patients and allowing for focused investigation on different categories of traumatic brain injury. Additional patient demographics, such as ethnicity, education level, and socioeconomic status could be collected to reflect social determinats of functional outcome. Furthermore, data quality may be improved by consistent, standardized formal training of FIM score evaluators. Although machine learning prediction algorithms are positioned to surpass expert interpretation and traditional statistical analysis, the models remain vulnerable to the inherent variability, quality, and bias based on the training data.

## Conclusion

Machine learning statistical models and artificial intelligence may accurately provide valuable, granular clinical predictions of neurologic outcomes for TBI patients receiving rehabilitation services. Our study is the first to apply machine learning statistical algorithms to this population, TBI patients receiving acute rehabilitation. We found that acute rehabilitation services significantly improve FIM scores for TBI patients and ultimately, their clinical recovery and reintegration into society.

## Data Availability

The datasets presented in this article are not readily available because Dataset held privately by rehabilitation facility. Requests to access the datasets should be directed to MZSun@mednet.ucla.edu.
